# Bias Stress Stability of Solution-Processed Nano Indium Oxide Thin Film Transistor

**DOI:** 10.3390/mi12020111

**Published:** 2021-01-22

**Authors:** Rihui Yao, Xiao Fu, Wanwan Li, Shangxiong Zhou, Honglong Ning, Biao Tang, Jinglin Wei, Xiuhua Cao, Wei Xu, Junbiao Peng

**Affiliations:** 1 State Key Laboratory of Luminescent Materials and Devices, Institute of Polymer Optoelectronic Materials and Devices, South China University of Technology, Guangzhou 510640, China; yaorihui@scut.edu.cn (R.Y.); 201630343721@mail.scut.edu.cn (X.F.); mswanwanli@mail.scut.edu.cn (W.L.); 201820117973@mail.scut.edu.cn (S.Z.); magicwei@foxmail.com (J.W.); xuwei@scut.edu.cn (W.X.); psjbpeng@scut.edu.cn (J.P.); 2 Guangdong Provincial Key Laboratory of Optical Information Materials and Technology & Institute of Electronic Paper Displays, South China Academy of Advanced Optoelectronics, South China Normal University, Guangzhou 510006, China; biao.tang@guohua-oet.com; 3 State Key Laboratory of Advanced Materials and Electronic Components, Fenghua Electronic Industrial Park, No. 18 Fenghua Road, Zhaoqing 526020, China

**Keywords:** indium oxide thin film, solution method, plasma surface treatment, annealing temperature, bias stability

## Abstract

In this paper, the effects of annealing temperature and other process parameters on spin-coated indium oxide thin film transistors (In_2_O_3_-TFTs) were studied. The research shows that plasma pretreatment of glass substrate can improve the hydrophilicity of glass substrate and stability of the spin-coating process. With Fourier transform infrared (FT-IR) and X-ray diffraction (XRD) analysis, it is found that In_2_O_3_ thin films prepared by the spin coating method are amorphous, and have little organic residue when the annealing temperature ranges from 200 to 300 °C. After optimizing process conditions with the spin-coated rotating speed of 4000 rpm and the annealing temperature of 275 °C, the performance of In_2_O_3_-TFTs is best (average mobility of 1.288 cm^2^·V^−1^·s^−1^, I_on_/I_off_ of 5.93 × 10^6^, and SS of 0.84 V·dec^−1^). Finally, the stability of In_2_O_3_-TFTs prepared at different annealing temperatures was analyzed by energy band theory, and we identified that the elimination of residual hydroxyl groups was the key influencing factor. Our results provide a useful reference for high-performance metal oxide semiconductor TFTs prepared by the solution method.

## 1. Introduction

With the active matrix liquid crystal display (AMLCD) and active matrix organic light-emitting diodes (AMOLED) gradually occupying the mainstream position in the display field [1,2,3,4], metal oxide thin film transistors (MOS-TFTs) have been widely studied due to their high mobility, high light transmittance, low processing temperature and low processing cost [3,4,5,6,7,8,9]. At present, metal oxide semiconductors are mainly fabricated by vacuum deposition methods, which have strict environmental requirements and relatively high manufacturing cost [10,11]. In contrast, solution-processed deposition offers the advantages of a simple process, high-throughput, high material utilization rate, and easy control of chemical components, which provides the possibility for large-area preparation of metal oxide semiconductor [12,13,14,15,16,17]. The preparation of metal oxides by the solution method usually requires annealing, which promotes the formation of (M–O–M) structure and the densification of film [18]. Solution methods mainly include the spin-coating method, solvothermal method, microwave assisted growth method, sonochemical method, hydrothermal method, electrodeposition method and so on [19,20,21,22,23,24,25]. Among them, the spin-coating method has the advantages of low cost, low pollution, energy saving and low film thickness, but it also has the disadvantages of uneven film thickness and waste of solution.

Nowadays, metal oxide semiconductor materials with (*n* − 1)d^10^ns^0^ (*n* ≥ 4) electronic configurations have attracted much attention due to their good electrical properties in the amorphous phase, and indium oxide (In_2_O_3_) is one of them [26,27]. This meets the requirements of low temperature preparation in the field of flexible display. In_2_O_3_ has been widely studied in the preparation of TFT active layer due to its wide band gap, high mobility, high carrier concentration and good transparency [1,28,29,30,31], but there is still little research on the influence mechanism of amorphous In_2_O_3_ electrical stability [32].

In this paper, In_2_O_3_ thin films were prepared by spin coating. The spreading property of precursor solution was improved by plasma surface treatment [33]. The phase composition of In_2_O_3_ thin films was investigated by different annealing methods. On this basis, In_2_O_3_-TFTs were fabricated on Al electrodes and Si_3_N_4_ substrates and the electrical characteristics of devices under different process conditions were analyzed. Furthermore, the possible methods to further improve the performance of TFTs prepared by spin coating were explored.

## 2. Materials and Methods

**Figure 1:**
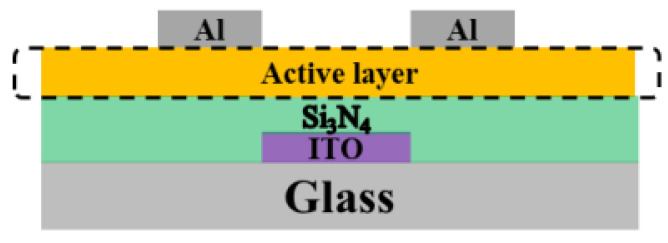
Schematic of indium oxide thin film transistors (In_2_O_3_-TFTs).

A semiconductor precursor solution was prepared using Indium nitrate hydrate (In(NO_3_)_3_·5H_2_O, CAS No.: 13465-14-0), in a solvent of ethylene glycol monomethyl ether (2-MOE, CAS No.: 109-86-4). The precursor solution was stirred in a magnetic mixer for 30 min and filtered, and then ultrasonic treatment was performed for 10 min. After plasma surface treatment, 35 μL of In_2_O_3_ precursor solution was added dropwise to the single-crystal silicon (CAS No.: 7440-21-3), and then spin coated with a homogenizer (model: KW-4A) at the speed of 4000 rms for 30 s. In the multilayer spin-coating process, every single-layer was pre-annealed at 100 °C for 10 min to evaporate the solvent. Finally, the In_2_O_3_ films were annealed at 200 °C, 225 °C, 250 °C, 275 °C and 300 °C for 45 min respectively. For the fabrication of In_2_O_3_-TFTs, bottom-gate devices were fabricated on Al (CAS No.: 7429-90-5) gate electrode and Si_3_N_4_ (CAS No.: 27198-71-6) dielectric layer. After the deposition of In_2_O_3_ thin films, an Al source and drain electrodes were formed by magnetron sputtering through a shadow mask. The schematic structure of the In_2_O_3_-TFTs is shown in Figure 1.

The surface tension of In_2_O_3_ solutions were measured by an Attension Theta Lite (TL200, Biolin Scientific, Gothenburg, Sweden). The internal chemical composition of In_2_O_3_ thin films was observed by Fourier transform infrared spectroscopy (FT-IR) (IRprestige21, Shimadzu, Kyoto, Janpan). X-ray reflectivity (XRR) (EMPYREAN, PANalytical, Almelo, The Netherlands) was used to analyze the thickness of the films. X-ray diffraction (XRD) (EMPYREAN, PANalytical, Almelo, The Netherlands) was used to analyze the phase of the films with Cu-kα as the X-ray source, and the scanning speed is 0.1°·s^−1^ from 20° to 70°. The surface morphology of the films was measured by atomic force microscopy (AFM) (Being Nano-Instruments BY3000, Being Nano-Instruments, Beijing, China). Semiconductor parameter analyzers (Agilent 4155c, Agilent, Santa Clara, CA, USA) was used under an ambient atmosphere to evaluate the electrical characteristics of TFTs.

## 3. Results and Discussion

### 3.1. Effect of Plasma Surface Treatment on Solution Spreading

**Figure 2:**
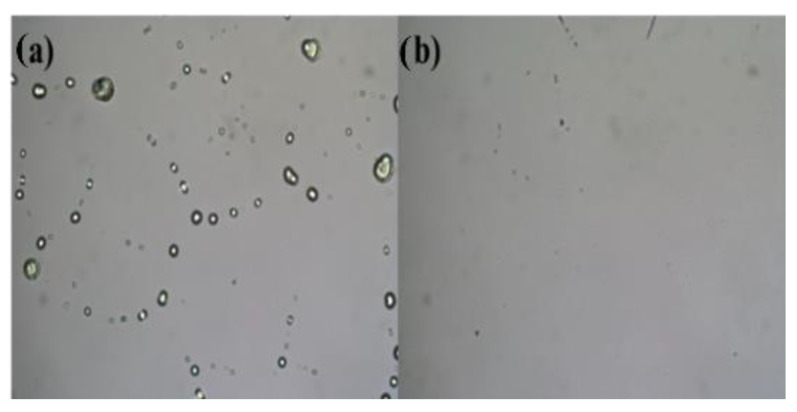
Spreading situation of precursor solution: (**a**) without plasma surface treatment; (**b**) with plasma surface treatment.

Figure 2a,b show the spread situation of In_2_O_3_ precursor solutions without/with plasma surface treatment. It can be found that it is difficult for the In_2_O_3_ precursor solution to spread uniformly on the substrate without plasma treatment. They agglomerate into many small droplets on the substrate, so the uniformity of the film is poor. However, on the substrate treated by plasma, the precursor solution of In_2_O_3_ can be spread uniformly without agglomeration, which indicates that plasma surface treatment can significantly improve the film-forming ability of spin coating. The results characterized by Attension Theta Lite show that the surface tension of In(NO_3_)_3_ solution is 39 mN·m^−1^, while that of 2-MOE is 27.6 mN·m^−1^, this difference easily leads to the spontaneous agglomeration of micro droplets after spin coating, so the film-forming ability is poor; however, plasma treatment can effectively reduce the difference of surface tension by introducing polarization groups into the surface of the substrate, thus improving the hydrophilicity of the substrate surface, which can improve the uniformity of spin-coating films [34,35,36]. Based on this result, the subsequent films and TFTs are prepared on substrates with plasma surface treatment.

### 3.2. Effect of Annealing Temperature on In_2_O_3_ Thin Films and TFTs

**Figure 3:**
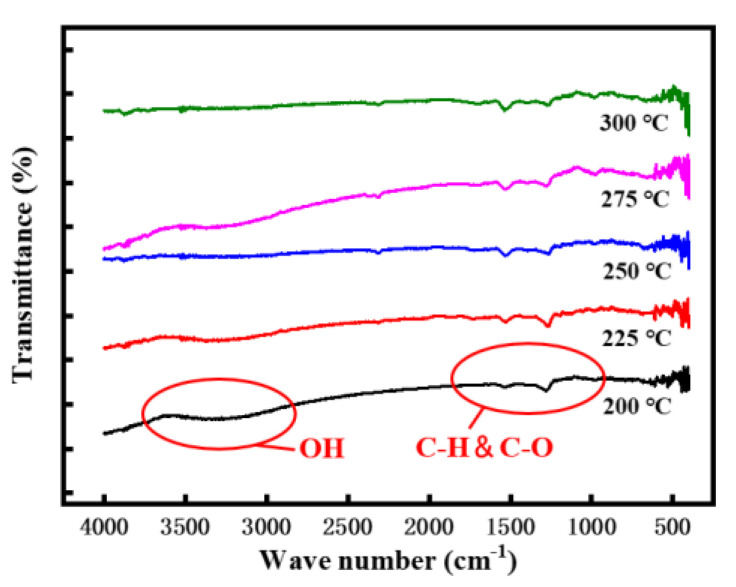
Fourier transform infrared (FT-IR) spectroscopy curves of In_2_O_3_ thin films annealed at different temperatures.

Figure 3 shows the FT-IR test curves of In_2_O_3_ thin films prepared at different annealing temperatures. The absorption peak located at 2750 cm^−1^ to 3750 cm^−1^ is the stretching vibration of O–H bond [37], The absorption peak at 1250 cm^−1^ to 1750 cm^−1^ were caused by the bending vibration of the carbon–hydrogen bond and carbon–oxygen bond [38]. The absorption peak at 500–700 cm^−1^ can be attributed to the stretching vibration of the In–O bond. The O-H bond mainly comes from [In (OH)]_(3−x)_^+^, and the hydrolysis reaction of precursor is as follows [18]:

In^3+^ + xH_2_O→[In(OH)]^(3−x)+^ + xH^+^ (1)

where x is the stoichiometric number.

Excessive concentration of such functional groups easily causes adverse effects on the electrical properties of TFTs, such as high leakage current. It can be seen from Figure 3 that with the increase of annealing temperature, the vibration peak intensity of the O–H bond decreases significantly, which is almost zero at 300 °C. Based on the discussion above, an annealing temperature higher than 250 °C is necessary for promoting metal-oxide bond formation. Combined with the process requirements of device preparation, such as preventing the defects from the hillock of the Al electrode, the appropriate annealing temperature range of In_2_O_3_ thin films is 250 °C to 300 °C.

**Figure 4:**
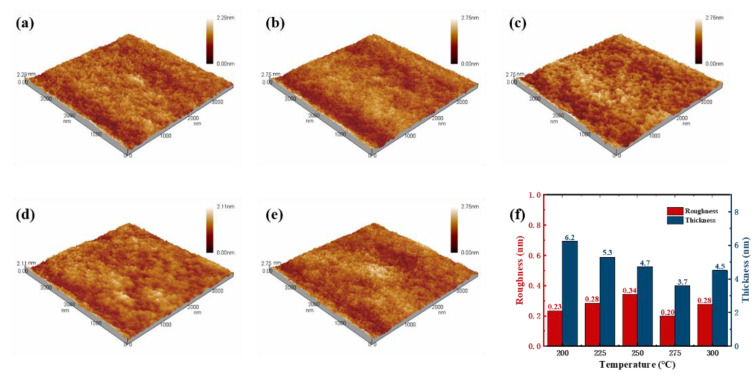
Atomic force microscopy (AFM) 3D images (4.0 × 4.0 µm^2^) of In_2_O_3_ thin films annealed at different temperatures: (**a**) 200 °C, (**b**) 225 °C, (**c**) 250 °C, (**d**) 275 °C, (**e**) 300 °C, respectively. (**f**) The surface roughness and thickness of these films.

To investigate the influence of the annealing temperature on the surface morphology and thickness of In_2_O_3_ thin films, AFM and XRR was used. The AFM scanning area of the images was 4.0 µm × 4.0 µm. Figure 4a–e shows AFM images of In_2_O_3_ thin films annealed at different temperature, and it can be seen that all samples show a relatively smooth morphology without cracks with low roughness (below 0.4 nm). At the same time, the thickness of In_2_O_3_ films at different annealing temperatures are in the range of 3–7 nm, which indicates that they are all nano-scale films.

**Figure 5:**
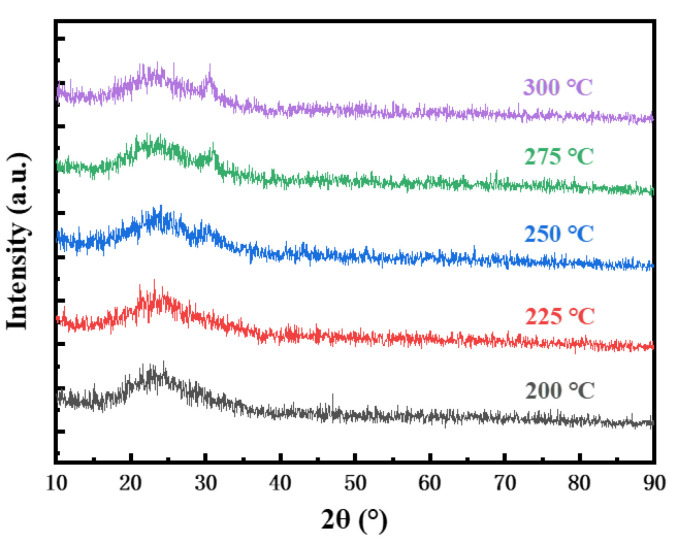
X-ray diffraction (XRD) curves of In_2_O_3_ annealed at different temperatures.

In our previous studies, it was found that In_2_O_3_ thin films crystallized only at temperatures above 400 °C [17]. Therefore, we only studied the crystallization of In_2_O_3_ thin films at 200–300 °C in this work. It can be seen from Figure 5 that as the annealing temperature increases, a diffraction peak related to cubic In_2_O_3_ gradually appears in the film around 31°, which indicates an increased transformation from amorphous phase into crystalline phase. According to the test results, when the temperature is 275 °C and below, the diffraction peak has a large full width at half maxima (FWHM), weak intensity, and extremely low crystallinity, which indicates that the In_2_O_3_ thin films prepared by the spin coating can maintain an amorphous structure at this annealing temperature. The amorphous In_2_O_3_ thin films can achieve better flatness and uniformity than crystalline films, and is conducive to the control of carrier concentration [38].

**Figure 6:**
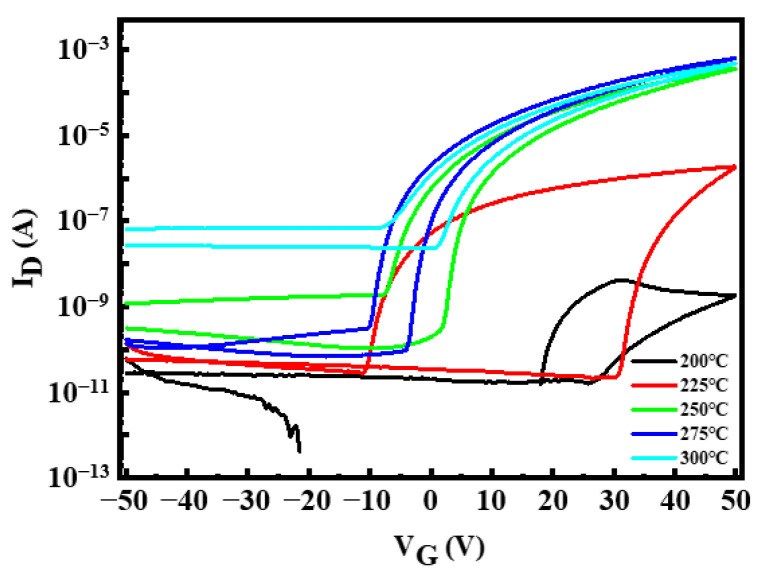
Transfer current-voltage (I-V) characteristics of In_2_O_3_-TFTs at different annealing temperature.

In_2_O_3_-TFTs were prepared under the optimized conditions. Figure 6 shows the transfer I–V characteristics of In_2_O_3_-TFTs at different annealing temperatures, and their corresponding electrical characteristics are listed in Table 1. It can be seen from Figure 6 that the devices had a certain negative shift and a large hysteresis, which indicated that there were many defects in the active layer. Combined with the test results in Figure 3, it may be the residual OH^-^ in the In_2_O_3_ films. Another possible factor for the results is that H+ in the Si_3_N_4_ gate insulating layer. With the increase of annealing temperature, the hydroxyl group gradually decomposes, and the hysteresis phenomenon gradually weakens [39]. When the annealing temperature is 275 °C, the threshold voltage (V_th_) difference between forward scanning and reverse scanning is the smallest, and the In_2_O_3_-TFTs showed a V_th_ of 0.84 V, an I_on_/I_off_ ratio of 5.93 × 10^6^, which was ideal.

It can be seen from Table 1 that the V_th_ of In_2_O_3_-TFTs gradually negative shift with the increase of annealing temperature, which is the minimum (0.84 V) at 275 °C. Also, the I_on_/I_off_ first increased to a peak value (5.93 × 10^6^) at 275 °C, and then decreased. Furthermore, the saturation mobility (μ_sat_) and subthreshold swing (SS) reached the maximum (1.288 cm^2^·v^−1^·s^−1^) and the minimum (1.030 V·dec^−1^) at 275 °C respectively. The performance is similar to that of In_2_O_3_-TFTs prepared by Choi at 280 °C (μ_sat_ of 2.4 cm^2^·v^−1^·s^−1^, I_on_/I_off_ of 10^6^) [40]. According to the FT-IR results, the low carrier concentration inside the In_2_O_3_ films at low annealing temperature may be due to the existence of undecomposed metal hydroxides in the active layer in the form of various defects, leading to the low μ_sat_, low I_on_/I_off_, large V_th_ and large SS. When the annealing temperature increased from 200 °C to 275 °C, the devices performance gradually improved. However, when the annealing temperature reached 300 °C, the carrier concentration in the In_2_O_3_ thin films was too high, which made the device unable to turn off normally, thus, the I_on_/I_off_ was only 7.27 × 10^3^. In addition, the continuous increase of temperature may lead to the degradation of the interface quality of the device, leading to the decrease of other performance.

### 3.3. Bias Stability of Indium Oxide Thin Film Transistors (In_2_O_3_-TFTs)

**Figure 7:**
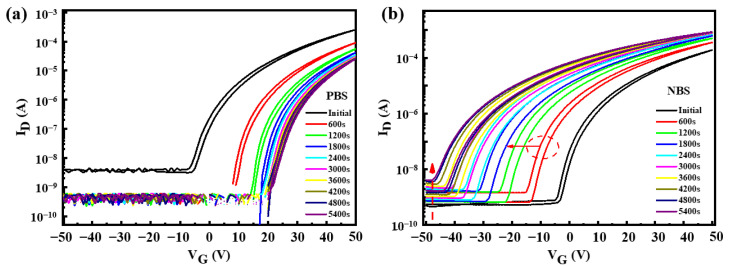
Transfer curves of In_2_O_3_-TFT annealed at 275 °C under (**a**) positive gate bias stress (PBS) and (**b**) negative gate bias stress (NBS). Measurement conditions: V_DS_ = 20 V at room temperature.

**Figure 8:**
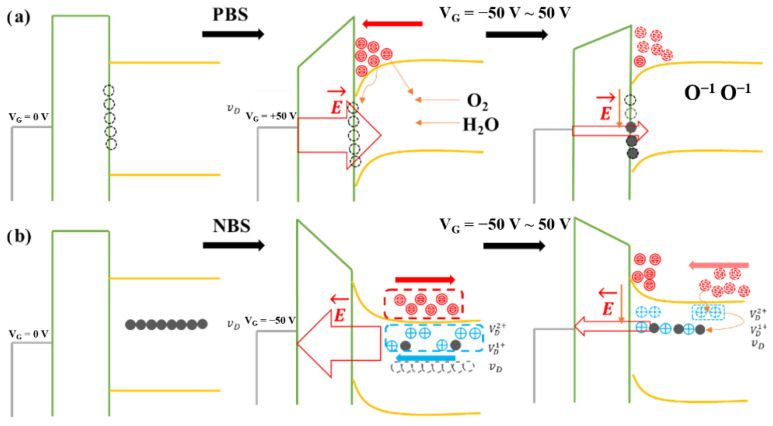
Plot of how about the energy band and carriers of In_2_O_3_-TFTs changing with the electric field under the (**a**) PBS and (**b**) NBS.

Figure 7 shows the transfer curves of In_2_O_3_-TFTs annealed at 275 °C under positive gate bias stress (PBS) and negative gate bias stress (NBS) with a drain-bias stress of V_DS_ = 20 V. During the test, a bias stress (V_GS_ = −50 V for NBS and V_GS_ = 50 V for PBS) was applied to the gate electrode for 5400 s. Figure 8 shows the energy band change of In_2_O_3_-TFTs under bias voltage. It can be seen from Figure 7a that the V_th_ under PBS drifts forward nearly 20 V. The possible reasons are as follows: (1) the carriers were trapped by the interface defects of In_2_O_3_/Si_3_N_4_; (2) the In_2_O_3_ back channel adsorbed water and oxygen in the environment, and oxygen atoms captured electrons [41]. In both cases, as shown in Figure 8a, the actual carrier concentration will be reduced, so a higher V_th_ is required to form the conductive channel.

Similarly, V_th_ had a drift of −45 V under NBS, as shown in Figure 7b. In addition, with the increase of NBS time, the I_off_ increased and the hysteresis phenomenon became more obvious, which indicated that there were more defects in the In_2_O_3_ active layer, or the adsorption of water and oxygen was more serious. This was due to the activation of a large number of donors under NBS, such as impurities in the In_2_O_3_ films, and these electrons were removed from the active layer by the electric field. At the same time, the donors were positively charged after losing electrons, which were absorbed near the In_2_O_3_/Si_3_N_4_ interface. When the bias voltage changed from −50 V to 0 V, the electric field cannot remove all the electrons generated out of the channel. Also, most of the electrons were not easy to compound with donor-like vacancies, but directly formed channel current under voltage, resulting in a negative V_th_, as shown in Figure 8b.

## 4. Conclusions

In this study, we fabricated solution-processed In_2_O_3_ thin films and TFTs, and investigated the factors affecting the stability of the devices. The results show that plasma treatment can significantly improve the spreading of In_2_O_3_ precursor solution on the substrate surface. The In_2_O_3_ films without annealing contain more organic residues, and they can be significantly reduced after annealing at 275 °C maintaining the amorphous film structure.

Through the study of the annealing characteristics of In_2_O_3_-TFTs, it was found that the devices prepared by the solution method have the characteristics of low active layer carrier concentration, high V_th_ and low I_on_/I_off_ at low temperature. With the increase of annealing temperature, the electrical properties of the devices gradually improve. The optimal performance can be obtained after annealing at 275 °C, which exhibited a high μ_sat_ of 1.288 cm^2^·V^−1^·s^−1^, high I_on_/I_off_ of 5.93 × 10^6^, and low SS of 0.84 V·dec^−1^. Also, the bias stability is the best, which may be due to the reduction of organic residues and defects in the films. When further increasing the annealing temperature, the performance deteriorates, which may be due to the film interface degradation. This study provides a useful reference for the improvement and optimization of the performance of electronic devices prepared by the solution method.

## Figures and Tables

**Table 1:** Properties of In_2_O_3_-TFTs at different annealing temperature.

Temperature (°C)	V_th_ (V)	I_on_/I_off_	μ_sat_ (cm^2^·V^−1^·s^−1^)	SS (V·dec^−1^)
200	-	2.83 × 10^2^	-	-
225	39.67	6.70 × 10^4^	-	-
250	6.46	3.00 × 10^5^	0.837	1.77
275	0.84	5.93 × 10^6^	1.288	1.03
300	–2.93	7.27 × 10^3^	1.099	2.69
